# Sex differences in the relationship between olfactory and cognitive impairment among subjects with subjective cognitive decline and mild cognitive impairment

**DOI:** 10.1186/s13293-025-00691-x

**Published:** 2025-02-13

**Authors:** Qin Liu, Ben Chen, Qiang Wang, Danyan Xu, Mingfeng Yang, Gaohong Lin, Yijie Zeng, Jingyi Lao, Shuang Liang, Jiafu Li, Kexin Yao, Xiaomei Zhong, Yuping Ning

**Affiliations:** 1 The First School of Clinical Medicine, Southern Medical University, Guangzhou, Guangdong Province China; 2 Geriatric Neuroscience Center, The Affiliated Brain Hospital of Guangzhou Medical University, Mingxin Road #36, Liwan District, Guangzhou, 510370 Guangdong Province China; 3 Guangdong Engineering Technology Research Center for Translational Medicine of Mental Disorders, Guangzhou, China; 4 Key Laboratory of Neurogenetics and Channelopathies of Guangdong Province and the Ministry of Education of China, Guangzhou Medical University, Guangzhou, China

**Keywords:** Subjective cognitive decline, Mild cognitive impairment, Sex differences, Olfaction, Cognition, Moderating effect

## Abstract

**Background:**

Odor identification (OI) deficits are observed in both individuals with subjective cognitive decline (SCD) and mild cognitive impairment (MCI), and serve as risk factors for dementia. Compared with males, females typically demonstrate superior OI performance and different risks of dementia. However, the role of sex in the relationship between OI dysfunction and cognitive impairment remains uncertain.

**Methods:**

In total, 121 subjects with SCD (41 males and 80 females), and 169 subjects with MCI (59 males and 110 females) underwent the Sniffin’ Sticks Screen 16 test and comprehensive neuropsychological examination. The relationships between olfactory and cognitive impairment were analyzed via partial correlation, multiple linear regression and moderating effects.

**Results:**

In both SCD and MCI subjects, males performed better in language and females performed better in memory. The correlation between OI and cognition tended to be stronger in MCI subjects than in SCD subjects. In MCI subjects, the correlation tended to be stronger in females. For MCI females, better OI performance was correlated with higher short-term memory and attention scores. For MCI males, better OI performance was correlated with higher short-term memory scores. The OI was correlated with language in SCD males and with attention in SCD females. Sex played a moderating role in the relationship between OI dysfunction and language in MCI subjects and the relationship between OI dysfunction and short-term delayed recall memory and language in SCD subjects.

**Conclusion:**

These findings revealed significant sex differences between OI dysfunction and cognitive impairment in SCD and MCI subjects. Sex differences should be considered when utilizing OI in clinical settings to predict cognitive function.

**Supplementary Information:**

The online version contains supplementary material available at 10.1186/s13293-025-00691-x.

## Plain language summary

Compared with males, females are at greater risk of dementia, and their olfactory abilities often demonstrate marked sex discrepancies that favor females. In this study, we investigated sex differences in olfaction and cognitive impairment and the role of sex in the relationship between olfactory and cognitive impairment. Because subjects with both subjective cognitive decline and mild cognitive impairment have olfactory impairment, we recruited a sample of 121 subjects with subjective cognitive decline (41 males and 80 females) and 169 subjects with mild cognitive impairment (59 males and 110 females) from the Affiliated Brain Hospital of Guangzhou Medical University and the community. We measured their olfaction and cognition. We found a positive correlation between olfaction and memory in mild cognitive impairment males, and olfaction was positively correlated with memory and attention in mild cognitive impairment females. Furthermore, sex served as a moderating factor in the relationship between olfaction and cognition among subjects with both subjective cognitive decline and mild cognitive impairment. Olfactory impairment may be helpful in predicting cognitive impairment in mild cognitive impairment subjects, and needs further follow-up, which is of great clinical importance for the use of olfaction to predict dementia in the future.

**Supplementary Information:**

The online version contains supplementary material available at 10.1186/s13293-025-00691-x.

## Highlights

- Females tended to have a stronger relationship between olfaction and cognition in MCI subjects.
- Olfactory performance was related to memory in MCI males, whereas it was related to memory and attention in MCI females.
- Sex played a moderating role in the relationship between olfaction and cognition in both SCD and MCI subjects.
- Our findings help elucidate the mechanisms of olfactory and cognitive impairment in males and females with SCD or MCI.

**Supplementary Information:**

The online version contains supplementary material available at 10.1186/s13293-025-00691-x.

## Background

As the older adult population continues to expand at a rapid pace worldwide, dementia has become a focal point of attention owing to its high occurrence in this demographic and the significant societal impact it has. Alzheimer’s disease (AD) is the predominant type of dementia, potentially accounting for 60–70% of all cases [1]. There is still no cure for this disease, and early prediction remains essential. Diagnostic biomarkers include amyloid-β (Aβ) and tau [2], however, cerebrospinal fluid (CSF) [3] acquisition is an invasive process, and positron-emission tomography (PET) [4] is expensive and limited by the application of Aβ and tau tracers. Therefore, finding noninvasive, simple, and economical markers would facilitate mass adoption.

Ageing is often accompanied by a decline in odor capabilities [5, 6], but severe olfactory loss observed in patients with dementia [7]. Subjective cognitive decline (SCD) is the preclinical stage of dementia [8, 9], whereas mild cognitive impairment (MCI) is an early stage of dementia [10], and both have olfactory impairments [11, 12]. Olfactory capacity [13] is evaluated through tests that measure threshold, discrimination, identification, pleasantness, familiarity and other odors. According to several studies, the olfactory identification test alone can serve as a screening tool for olfactory dysfunction [14] and studies have proven that odor identification dysfunction can predict cognitive decline [11, 15, 16].

Sex differences are known to occur in the course of AD, as well as in olfaction. Studies have shown that more females have MCI and AD [17, 18]. Researchers using data from the French National Alzheimer Database (BNA) [19] reported that females presented a 2–3% greater likelihood of transitioning from MCI to AD. Numerous studies have explored sex differences in olfactory ability. The National Geographic Smell Survey (NGSS) [20] involving 1.2 million people revealed that females were able to correctly identify more odors. The Olfaction in Catalonia (LOFACAT) [21] survey investigated olfaction in the general population, based on a study of 9348 surveys of both sexes and all ages, researchers revealed that olfaction was better in females than in males. However, the sex-related associations between olfaction and cognition in subjects with SCD and MCI remain uncertain.

Therefore, the aims of the current study were to explore (1) sex differences in olfaction and cognition in subjects with MCI and SCD; (2) sex differences in the correlation of olfaction and cognition in MCI and SCD subjects; and (3) whether sex moderates the relationship between olfaction and cognition in MCI and SCD subjects. This study provides a deeper understanding of the effects of sex on olfaction and cognition in MCI and SCD subjects to guide the use of olfaction to predict cognition.

## Methods

### Subjects

A total of 290 subjects (169 with MCI, 121 with SCD) were continuously recruited from the Affiliated Brain Hospital of Guangzhou Medical University and the community in Guangzhou. All subjects or their legal guardians provided signed informed consent to participate in the study. The ethics committees of the Affiliated Brain Hospital of Guangzhou Medical University approved this study.

The diagnostic criteria for MCI were based on the Peterson criteria [22]. The SCD criteria include two major features [23]: a self-experienced persistent decline in cognitive capacity relative to a previously normal cognitive status unrelated to an acute event, and after adjusting for age, sex and years of education, compared with normal controls, the score difference of each test in the neuropsychological battery was less than 1.5 standard deviations. All the subjects underwent structured interviews, standardized olfactory tests, and clinical symptom and comprehensive cognitive assessments.

### Assessment of cognitive function

All the subjects were interviewed by neuropsychologists to assess global cognitive function using Mini-Mental State Examination (MMSE, 30 points, > 24 as normal) [24], five cognitive domains including memory (Auditory Verbal Learning Task, AVLT) [25], language (Boston Naming Test, BNT [26], 30 points, > 22 as normal and Verbal Fluency Test, VFT [27], > 10 as normal), executive function (Stroop Color and Word Test C, Stroop C [28], with scores based on how many seconds were required to complete the exam), visuospatial skill (Rey-Osterrieth Complex Figure Test, ROCF [29], 36 points, > 30 as normal) and attention (Symbol-Digit Modality Test, SDMT [30], > 28 as normal, Digit Span Test, DST [31], 24 points, > 19 as normal and Trail-Making Test A, TMT A [32], with scores based on how many seconds were required to complete the exam). The AVLT N1-3 (sum scores of AVLT N1, N2, and N3), AVLT N4, AVLT N5, and AVLT N6 represent immediate recall, short-term delayed recall, long-term delayed recall and recognition, respectively (36 points, 12 points, 12 points, and 24 points, respectively).

### Assessment of olfactory function

For OI assessment, the Sniffin’ Sticks Screen 16 test [13] was applied, which involves the presentation of odorants from felt-tip pens. To measure OI performance, odorized pens were used. The pen’s cap was opened by the experimenter for approximately 3 s, and the pen’s tip was placed approximately 2 cm in front of both nostrils. The subjects were asked to smell 16 common odorants from the felt-tip pens and to name the odors using a multiple-choice format with 4 choices, only 1 of which was correct. The subjects’ scores ranged from 0 to 16. An OI score less than 10 was defined as OI dysfunction [33].

### Data analysis

Demographic and clinical data analysis and data visualization were done by using SPSS 25.0 (SPSS, Chicago, IL, USA), R version 4.3.2 (R Core Team, R Foundation for Statistical Computing, Vienna, Austria) and RStudio 2023.09.1 (RStudio Team. RStudio Inc., Boston, MA, USA). The differences in demographic, clinical, cognitive and olfactory information between males and females were analyzed using T-test for two independent samples, Mann–Whitney U test or χ^2^ test. Partial correlations were used to investigate the correlation between the cognitive scores and the values of OI, controlling variables included age, years of education. To investigate whether the factors influencing OI differed between males and females, we used multiple linear regression analysis, with the variables from the partial correlation analysis. The Hayes PROCESS macro was used to assess the moderating effect of sex on the relationship between OI dysfunction and cognitive function. OI was the predictor variable, cognition was the outcome variable, age and years of education were the covariates.

## Results

### Demographic, clinical, cognitive and olfactory information

In this study, a total of 290 people, 169 with MCI (59 males and 110 females), and 121 with SCD (41 males and 80 females) were included. As shown in Table 1, males had significantly higher BNT scores (*p* < 0.05) than females for both MCI and SCD subjects. In the population, females were more likely to have higher AVLT scores, including N1-3, N4, N5, and N6 scores (*p* < 0.05) in MCI females, and with the exception of AVLT N6 scores, the remaining AVLT scores were also higher in SCD females. No sex differences in demographic, clinical or olfactory information were observed.

**Table 1:** Demographic data, clinical information, cognitive function, and olfactory function of male and female MCI and SCD subjects

	MCI (n = 169)				SCD (n = 121)			
	Male (n = 59)	Female (n = 110)	t/Z/χ^2^	*p*-value	Male (n = 41)	Female (n = 80)	t/Z/χ^2^	*p*-value
Age	68.90 ± 9.33	67.97 ± 8.05	− 0.673	0.502	67.05 ± 5.99	67.91 ± 6.31	0.725	0.470
Years of education	9.53 ± 3.40	8.63 ± 3.46	− 1.172	0.241	12.02 ± 3.30	11.18 ± 2.87	− 1.644	0.100
OI score	9.17 ± 2.76	9.95 ± 2.31	− 1.679	0.093	10.90 ± 2.38	10.65 ± 2.09	− 0.748	0.455
OI dysfunction (%)	29 (49.15%)	41 (37.27%)	2.234	0.135	10 (24.39%)	21 (26.25%)	0.049	0.824
BMI	23.21 ± 2.98	22.99 ± 2.90	− 0.460	0.646	23.11 ± 3.02	23.04 ± 3.28	− 0.116	0.907
HIS	1.44 ± 0.68	1.65 ± 0.88	− 1.157	0.247	1.56 ± 0.78	1.71 ± 1.29	− 0.266	0.790
NPI	22.95 ± 1.14	22.33 ± 2.54	− 1.678	0.093	22.85 ± 1.53	22.84 ± 1.48	− 0.394	0.693
Global cognition								
MMSE	23.03 ± 3.48	23.81 ± 3.23	− 1.548	0.122	26.83 ± 1.86	26.60 ± 2.34	− 0.161	0.872
Memory								
AVLT N1-3	14.02 ± 4.62	15.87 ± 5.77	2.130*	**0.035**	18.41 ± 4.02	20.43 ± 5.00	2.229*	**0.028**
AVLT N4	3.83 ± 2.39	4.85 ± 2.61	− 2.314*	**0.021**	6.59 ± 1.92	7.40 ± 2.30	− 2.100*	**0.036**
AVLT N5	2.66 ± 2.40	3.80 ± 2.88	− 2.424*	**0.015**	5.95 ± 1.66	6.87 ± 2.61	− 2.431*	**0.015**
AVLT N6	2.54 ± 2.58	3.60 ± 2.73	− 2.538*	**0.011**	6.15 ± 2.09	6.99 ± 2.52	1.835	0.069
Language								
BNT	19.83 ± 3.55	18.25 ± 4.11	− 2.407*	**0.016**	24.24 ± 2.22	22.67 ± 2.86	− 3.245**	**0.001**
VFT	5.87 ± 1.94	6.45 ± 2.36	1.331	0.186	7.79 ± 2.63	8.32 ± 2.16	1.048	0.298
Executive function								
Stroop C	41.39 ± 7.51	42.97 ± 6.43	− 0.891	0.373	46.15 ± 4.79	46.43 ± 3.58	− 0.082	0.935
Visuospatial skill								
ROCF	23.96 ± 6.77	22.31 ± 6.96	− 1.902	0.057	27.67 ± 4.37	27.54 ± 4.18	− 0.156	0.876
Attention								
SDMT	26.21 ± 9.64	28.57 ± 11.68	1.301	0.195	35.15 ± 8.68	37.65 ± 8.81	1.483	0.141
DST	8.75 ± 2.26	8.81 ± 2.38	− 0.313	0.754	10.05 ± 2.01	10.24 ± 1.85	− 0.345	0.730
TMT A	62.07 ± 25.63	64.29 ± 33.66	− 0.113	0.910	45.39 ± 16.90	44.95 ± 15.12	− 0.348	0.728

Bold means that the significant p values. p-value meant the comparison between men and women by T-test for two independent samples, Mann–Whitney U test or χ^2^ test. Continuous variables are reported as mean ± standard deviation. * Statistically significant at the 0.05 level (2-tailed); ** Statistically significant at the 0.01 level (2-tailed)

MCI, mild cognitive impairment; SCD, subjective cognitive decline; OI, odor identification; OI dysfunction (%), percentage of OI dysfunction; BMI, Body Mass Index; HIS, Hachinski Inchemic Score; NPI, Neuropsychiatric Inventory; MMSE, Mini-Mental State Examination; AVLT N1-3, Auditory Verbal Learning Test Immediate recall; AVLT N4, Auditory Verbal Learning Test Short-term delayed recall; AVLT N5, Auditory Verbal Learning Test Long-term delayed recall; AVLT N6, Auditory Verbal Learning Test Recognition; BNT, Boston Naming Test; VFT, Verbal Fluency Test; The Stroop Color and Word Test; Stroop: The Stroop Color and Word Test; ROCF, Rey-Osterrieth Complex; SDMT, Symbol-Digit Modality Test. DST, Digit Span Test; TMT, Trail-Making Test.

### Partial correlation analyses

**Fig. 1:**
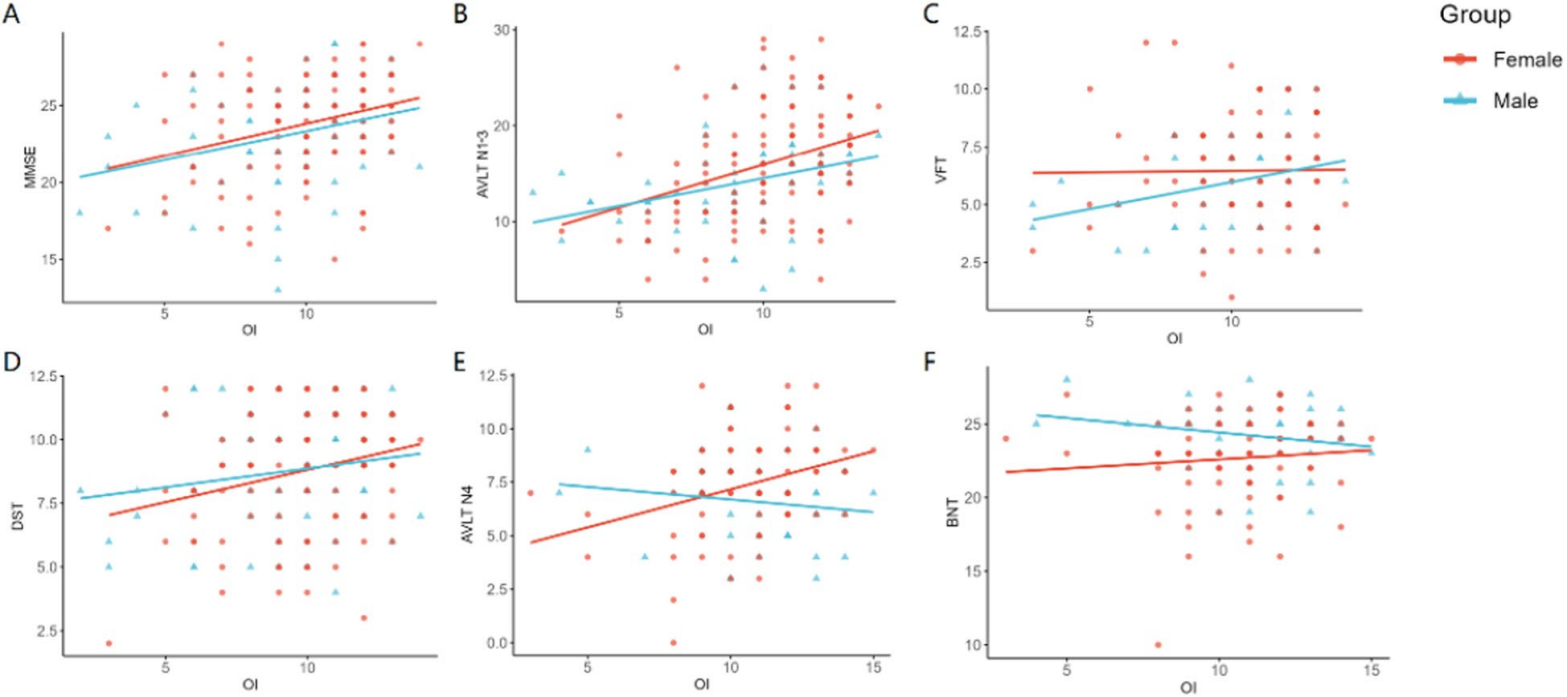
Partial correlation between OI and other variables (control variables included age, years of education). **A** OI was associated with MMSE in MCI females (r = 0.299, p = 0.002) and MCI males (r = 0.294, p = 0.029). **B** OI was associated with AVLT N1-3 in MCI females (r = 0.349, p < 0.001) and MCI males (r = 0.353, p = 0.008). **C** OI was associated with VFT in MCI males (r = 0.372, p = 0.028) but not in MCI females. **D** OI was associated with DST in MCI females (r = 0.258, p = 0.007) but not in MCI males. **E** OI was not associated with AVLT N4 in SCD males or females. **F** OI was not associated with BNT in SCD males or females. MCI, mild cognitive impairment; SCD, subjective cognitive decline; OI, odor identification; MMSE, Mini-Mental State Examination; AVLT N1-3, Auditory Verbal Learning Test Immediate recall; AVLT N4, Auditory Verbal Learning Test Short-term delayed recall; VFT, Verbal Fluency Test; DST, Digit Span Test; BNT, Boston Naming Test

As shown in Table S1, after controlling for age and years of education, the OI score was found to be related to the MMSE, AVLT N1-3, AVLT N6, BNT, VFT and SDMT in males with MCI. Body mass index (BMI), neuropsychiatric inventory (NPI), MMSE, AVLT N1-3, AVLT N4, AVLT N5, BNT, Stroop C, TMT A, ROCF, SDMT and DST were correlated with OI score in MCI females. In SCD subjects, the OI score was associated with VFT in males, and SDMT in females. The correlations between males and females are shown in Fig. 1.

### Multiple linear regression

For males with MCI, the model p = 0.006 indicated that this regression equation was valid. The model adjusted R^2^ was 0.164, indicating that MMSE, AVLT N1-3, AVLT N6, BNT, VFT, and SDMT could explain 16.4% of the change in OI score. AVLT N1-3 (B = 0.279, *p* = 0.006) had a positive effect on OI score, whereas the remaining variables had no significant effect. For females with MCI, the regression analysis between clinical information, cognition and the OI score revealed a significant relationship between the OI score and AVLT N1-3 (B = 0.143, *p* < 0.001), and DST (B = 0.192,* p* = 0.041), with an adjusted R^2^ of 0.177 (see Table 2).

**Table 2:** Results of the stepwise linear regression between OI score and cognitive impairment in MCI subjects

	B	Standard error	*P* value	Lower 95% CI	Upper 95 % CI	β
Male (n = 59)						
AVLT N1-3	0.279	0.096	0.006	0.085	0.474	0.432
Constant	5.248	1.545	0.002	2.116	8.379	
Adjusted R^2^			0.164			
Corrected p			0.012			
Female (n = 110)						
AVLT N1-3	0.143	0.038	< 0.001	0.068	0.219	0.348
DST	0.192	0.093	0.041	0.008	0.376	0.192
Constant	5.913	0.918	< 0.001	4.092	7.734	
Adjusted R^2^			0.177			
Corrected p			< 0.001			

B, Unstandardized coefficient; Std. error, standard error; CI, Confidence Interval; β, Standardized coefficient; Corrected p = p*2; AVLT N1-3, Auditory Verbal Learning Test Immediate recall; DST, Digit Span Test

### Moderating analyses

**Fig. 2:**
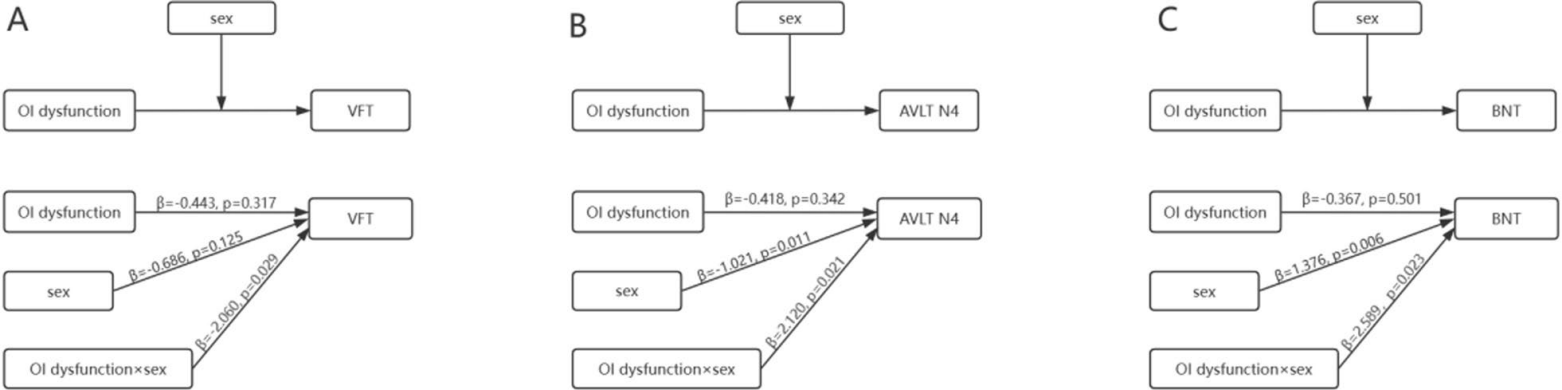
**A** Conceptual and statistical model of the association between OI dysfunction and VFT moderated by sex in MCI subjects. **B** Conceptual and statistical model of the association between OI dysfunction and AVLT N4 moderated by sex in SCD subjects. **C** Conceptual and statistical model of the association between OI dysfunction and BNT moderated by sex in SCD subjects

For the moderation analysis between OI dysfunction and cognitive function with age and years of education as covariates, the overall model (R^2^ = 0.099, *p* = 0.044) and the moderating effect of sex (β = − 2.060, *p* = 0.029) on the relationship between OI dysfunction and VFT were significant in MCI subjects (Table 3, Fig. 2A). In SCD subjects, the moderating effect of sex on the relationship between OI dysfunction and AVLT N4 (β = 2.120, *p* = 0.021, overall model R^2^ = 0.196, *p* < 0.001) was significant. Additionally, both the overall model (R^2^ = 0.207, *p* < 0.001) and the moderating effect of sex on the relationship between OI dysfunction and BNT (β = 2.589, *p* = 0.023) were significant (Table 4, Fig. 2B, C).

**Table 3:** Process moderating effect model in MCI subjects

Model	β	SE	T	p	95%Cl	
VFT						
Constant	6.357	1.761	3.610	< 0.001***	2.866	9.847
OI dysfunction	− 0.443	0.440	− 1.006	0.317	− 1.315	0.430
Sex	− 0.686	0.444	− 1.546	0.125	− 1.566	0.194
OI dysfunction*sex	− 2.060	0.929	− 2.216	0.029*	− 3.901	− 0.218
Age	− 0.018	0.025	− 0.700	0.485	− 0.068	0.033
years of education	0.124	0.065	1.910	0.059	− 0.005	0.254

*p < 0.05, ***p < 0.001; MCI, mild cognitive impairment; OI, odor identification; VFT, Verbal Fluency Test

**Table 4:** Process moderating effect model in SCD subjects

Model	β	SE	T	p	95%Cl	
Model 1: AVLT N4						
Constant	10.358	2.193	4.724	< 0.001***	6.015	14.701
OI dysfunction	− 0.418	0.437	− 0.955	0.342	− 1.284	0.449
Sex	− 1.021	0.393	− 2.601	0.011*	− 1.799	− 0.243
OI dysfunction*sex	2.120	0.908	2.335	0.021*	0.321	3.918
Age	-0.077	0.032	− 2.432	0.017*	− 0.139	− 0.014
Years of education	0.171	0.062	2.775	0.006**	0.049	0.293
Model 2: BNT						
Constant	17.800	2.722	6.539	< 0.001***	12.408	23.193
OI dysfunction	− 0.367	0.543	− 0.675	0.501	− 1.442	0.709
Sex	1.376	0.487	2.822	0.006**	0.41	2.341
OI dysfunction*sex	2.589	1.127	2.296	0.023*	0.355	4.822
Age	0.034	0.039	0.868	0.387	− 0.044	0.112
years of education	0.272	0.076	3.559	0.001**	0.121	0.424

*p < 0.05, **p < 0.01, ***p < 0.001; SCD, subjective cognitive decline; OI, odor identification; AVLT N4, Auditory Verbal Learning Test Short-term delayed recall; BNT, Boston Naming Test

## Discussion

To the best of our knowledges, the present work is the first to compare sex differences in the relationships between OI and cognition in Chinese individuals with SCD and MCI. The key discoveries were as follows. (1) The correlation between OI and cognition tends to be stronger in MCI subjects than in SCD subjects. For SCD subjects, OI was associated with language in males and with attention in females. (2) In MCI subjects, the correlation between olfaction and cognition tends to be stronger in females than in males. The OI was associated with global cognition, memory, language, and attention in males with MCI. In females with MCI, OI was associated with global cognition, memory, language, executive function, visuospatial skills, attention, BMI, and NPI. (3) A greater number of factors influencing olfaction were present in MCI females. The primary factor influencing olfaction in males with MCI was memory, whereas in females with MCI, it was memory and attention. (4) Sex moderated the association between OI dysfunction and language in subjects with MCI, and moderated the relationship between OI dysfunction and both memory and language in SCD subjects.

This study revealed sex differences in some tasks, with males performing better on BNT and females performing better on AVLT in the two groups. There was no difference in the other tasks. As expected, the finding on the AVLT is in line with previous studies showing better female performance in memory tasks [34, 35]. Females are usually thought to excel in verbal ability but we found that females did not have a significant advantage in VFT. The finding on verbal fluency is consistent with a previous study [36] that demonstrated similar performance in verbal fluency between both sexes among healthy older controls and MCI subjects (*p* > 0.05), although the sample sizes in the present study were generally larger (59 males and 110 females with MCI vs. 28 males and 15 females; 41 males and 80 females with SCD vs. 28 males and 23 females in the healthy control group). Additionally, males in this study exhibited significantly better performance in the language domain for BNT than females did, which is in line with the findings of some studies. In numerous normative studies assessing BNT, sex is associated with naming performance, with males outperforming females [37–39].

Zhong et al. [40] conducted a study on OI and cognition using 18 questions from the Montreal Cognitive Assessment (MoCA) in older Americans living in the community. They found that the association between OI and cognition was more pronounced in female with MCI than in males. Our study confirmed this finding, showing a stronger relationship between OI and cognition in females with MCI compared to males. However, the present study was conducted in Chinese individuals with SCD and MCI, and cognitive domains were measured via a number of representative scales. There is growing evidence that deficits in OI are associated with cognitive impairment in older adults [41, 42]. The association between OI and cognition was less pronounced in subjects with SCD than in those with MCI. One possibility to explain this finding may be that in the mild cognitive impairment stage, there is already an objective cognitive decline, whereas in the subjective cognitive decline stage, the subjects may only have subjective cognitive problems, so the relationship between olfaction and cognition was not as intimate in SCD subjects.

For MCI males, OI scores and memory scores were positively correlated, and for MCI females, higher memory and attention scores were related to higher OI scores. Many studies have reported that OI is significantly associated with memory [42–45]. However, our finding that OI performance predicts impairments in memory and attention in MCI females is novel. Olfaction and attention are closely related; the more concentrated the attention is, the stronger the sensitivity of olfaction [46], and olfactory information can reflexively guide visual attention [47]. Females among MCI subjects had higher mean OI scores, although the difference was not statistically significant, which might have led to the association between OI and attention in MCI females. Similarly, in SCD females, OI was associated with attention. Wang et al. [16] found that OI dysfunction worsens with increasing severity in the AD disease spectrum. Compared with MCI females, SCD females have less impairment of olfaction, which might explain why females with SCD also exhibited this association. In SCD males, OI was associated with language. Holly et al. [48] reported that language ability is a significant predictor of olfactory performance. In our study, males with SCD outperformed females in language tasks, which might partially account for the observed association in these males. Additionally, sex differences in brain connectivity and network organization, as well as the influence of sex hormones, could affect this relationship. Variations in brain networks between sexes may alter the processing of olfactory information, leading to associations between olfactory performance and different cognitive functions [49, 50]. The effects of sex hormones such as estrogen and testosterone on cognitive function and sensory processing have been extensively studied. Estrogen replacement therapy is believed to increase attention in females [51], whereas testosterone may inhibit the practice effect in verbal fluency tasks [52].

This study further investigated the impact of sex on the association between OI dysfunction and cognition in older Chinese individuals with SCD and MCI. Our findings showed that sex moderated the relationship between OI dysfunction and language in both groups; additionally, in SCD subjects, sex moderated the relationship between OI dysfunction and memory. Although our study did not reveal a difference in OI between males and females, there were sex differences in the relationship between OI and cognition. Sex plays an important role in language. Females are often considered to have an advantage in language skills, typically excelling in both comprehension and expression [53]. In semantic decision tasks, females tend to perform faster than males do [54]. However, some studies suggest that males may have advantages in specific language tasks, such as naming tests [37–39], and they show greater left frontal activation during semantic retrieval [55]. In language processing, males exhibit more inhibitory connections from the inferior frontal gyrus to the superior temporal gyrus (STG), whereas females show more inhibitory connections from the superior parietal lobule to the STG [54]. These differences may explain why sex moderates the relationship between olfactory performance and language. There are differences between sexes in the memory domain, with females having a slight advantage over males. During episodic memory tasks, females show more structural covariance connections than males do [56]. The brain activation patterns of males and females are notably different. When performing working memory tasks, females are predominantly activated in the left hemisphere, whereas males exhibit bilateral or right-sided activation patterns [57]. These differences may explain why sex plays a moderating role in olfactory impairment and memory.

There are several limitations in the present study. First, because the study was cross-sectional, it was unable to actively track changes in olfactory and cognitive functions, which need to be refined after the research. Second, the current evaluations did not consider olfactory thresholds or olfactory discrimination, and future research should undertake a thorough evaluation of olfactory function, encompassing olfactory identification, olfactory thresholds, and olfactory discrimination. Third, imaging, pathological and other markers are lacking, so our sample is based on the entire population, which may lead to a limited representation of subjects with MCI and SCD, and further imaging and biomarker data must be collected.

### Perspectives and significance

The current study demonstrated the importance of considering sex differences in the relationship between olfaction and cognition in subjects with SCD and MCI. These findings contribute to a deeper understanding of how sex influences the interplay between olfaction and cognition in older adult populations, and they also aid in understanding the mechanisms of olfactory and cognitive impairment across sexes.

## Supplementary Information

Additional file 1.

## Data Availability

No datasets were generated or analysed during the current study.
