# Supplementary material for: Sex differences in the relationship between olfactory and cognitive impairment among subjects with subjective cognitive decline and mild cognitive impairment
Source: Biol Sex Differ. 2025 Feb 13;16:12. doi: 10.1186/s13293-025-00691-x (PMC11827212; doi:10.1186/s13293-025-00691-x)
Supplement: Supplementary file 1 — Additional file 1. [file 13293_2025_691_MOESM1_ESM.docx]

**Supplementary information**

**Tabel S1**. Partial Correlation Analyses between OI and other variables (control variables included age, years of education)

|  | MCI (n=169) | | | | | | |  | SCD（n=121） | | | | | | |
| --- | --- | --- | --- | --- | --- | --- | --- | --- | --- | --- | --- | --- | --- | --- | --- |
|  | Male (n=59) | | |  | Female (n=110) | | |  | Male (n=41) | | |  | Female (n=80) | | |
|  | r | *p* | corrected p |  | r | *p* | corrected *p* |  | r | *p* | corrected *p* |  | r | *p* | corrected *p* |
| BMI | -0.245 | 0.074 | 0.630 |  | 0.244 | **0.012** | 0.110 |  | 0.036 | 0.828 | 1.000 |  | 0.211 | 0.067 | 1.000 |
| HIS | 0.146 | 0.288 | 0.290 |  | 0.182 | 0.060 | 0.120 |  | -0.055 | 0.738 | 1.000 |  | -0.113 | 0.323 | 1.000 |
| NPI | 0.170 | 0.215 | 0.860 |  | 0.206 | **0.032** | 0.160 |  | -0.009 | 0.959 | 1.000 |  | -0.091 | 0.427 | 1.000 |
| **Global cognition** | |  |  |  |  |  |  |  |  |  |  |  |  |  |  |
| MMSE | 0.294 | **0.029** | 0.350 |  | 0.299 | **0.002** | **0.030** |  | 0.005 | 0.977 | 0.980 |  | -0.067 | 0.558 | 1.000 |
| **Memory** |  |  |  |  |  |  |  |  |  |  |  |  |  |  |  |
| AVLT N1-3 | 0.353 | **0.008** | 0.130 |  | 0.349 | **0.000** | **0.000** |  | -0.047 | 0.777 | 1.000 |  | 0.132 | 0.251 | 1.000 |
| AVLT N4 | 0.213 | 0.119 | 0.830 |  | 0.269 | **0.005** | 0.070 |  | -0.145 | 0.378 | 1.000 |  | 0.206 | 0.070 | 1.000 |
| AVLT N5 | 0.243 | 0.074 | 0.630 |  | 0.205 | **0.033** | 0.130 |  | -0.179 | 0.275 | 1.000 |  | 0.059 | 0.606 | 1.000 |
| AVLT N6 | 0.326 | **0.015** | 0.220 |  | 0.187 | 0.052 | 0.160 |  | -0.138 | 0.402 | 1.000 |  | 0.137 | 0.231 | 1.000 |
| **Language** |  |  |  |  |  |  |  |  |  |  |  |  |  |  |  |
| BNT | 0.282 | **0.037** | 0.410 |  | 0.259 | **0.007** | 0.090 |  | -0.196 | 0.233 | 1.000 |  | 0.107 | 0.349 | 1.000 |
| VFT | 0.372 | **0.028** | 0.360 |  | 0.010 | 0.933 | 0.930 |  | -0.453 | **0.009** | 0.160 |  | -0.049 | 0.720 | 1.000 |
| **Executive function** | |  |  |  |  |  |  |  |  |  |  |  |  |  |  |
| Stroop C | 0.159 | 0.270 | 0.540 |  | 0.221 | **0.023** | 0.180 |  | -0.024 | 0.884 | 1.000 |  | 0.090 | 0.431 | 1.000 |
| **Visuospatial skill** | |  |  |  |  |  |  |  |  |  |  |  |  |  |  |
| ROCF | 0.264 | 0.051 | 0.510 |  | 0.250 | **0.009** | 0.100 |  | 0.009 | 0.955 | 1.000 |  | 0.082 | 0.477 | 1.000 |
| **Attention** |  |  |  |  |  |  |  |  |  |  |  |  |  |  |  |
| SDMT | 0.381 | **0.005** | 0.090 |  | 0.223 | **0.025** | 0.150 |  | 0.151 | 0.358 | 1.000 |  | 0.226 | **0.049** | 0.882 |
| DST | 0.211 | 0.121 | 0.730 |  | 0.258 | **0.007** | 0.090 |  | -0.075 | 0.648 | 1.000 |  | -0.049 | 0.669 | 1.000 |
| TMT A | -0.180 | 0.197 | 0.990 |  | -0.218 | **0.024** | 0.170 |  | -0.094 | 0.568 | 1.000 |  | -0.073 | 0.525 | 1.000 |

Bold means that the significant P values. MCI, mild cognitive impairment; SCD, subjective cognitive decline; OI, odor identification; BMI, Body Mass Index; HIS, Hachinski Inchemic Score; NPI, Neuropsychiatric Inventory; MMSE, Mini-Mental State Examination; AVLT N1-3, Auditory Verbal Learning Test

Immediate recall; AVLT N4, Auditory Verbal Learning Test Short-term delayed recall; AVLT N5, Auditory Verbal Learning Test Long-term delayed recall; AVLT N6, Auditory Verbal Learning Test Recognition; BNT, Boston Naming Test; VFT, Verbal Fluency Test; The Stroop Color and Word Test; Stroop: The Stroop Color and Word Test; ROCF, Rey-Osterrieth Complex; SDMT, Symbol-Digit Modality Test. DST, Digit Span Test; TMT, Trail-Making Test.
